# Comparative Transcriptome Analysis of *Henosepilachna vigintioctomaculata* Reveals Critical Pathways during Development

**DOI:** 10.3390/ijms25147505

**Published:** 2024-07-09

**Authors:** Yu-Xing Zhang, Yi-Kuan Wu, Hai-Hui Liu, Wen-Ze Li, Lin Jin, Guo-Qing Li

**Affiliations:** Education Ministry Key Laboratory of Integrated Management of Crop Diseases and Pests/State & Local Joint Engineering Research Center of Green Pesticide Invention and Application, Department of Entomology, College of Plant Protection, Nanjing Agricultural University, Nanjing 210000, China; 2020202042@stu.njau.edu.cn (Y.-X.Z.); 2021102077@stu.njau.edu.cn (Y.-K.W.); 2022102064@stu.njau.edu.cn (H.-H.L.); 2021102078@stu.njau.edu.cn (W.-Z.L.); jinlin@njau.edu.cn (L.J.)

**Keywords:** *Henosepilachna vigintioctomaculata*, transcriptome, unigene, developmental stage, differential expression genes

## Abstract

*Henosepilachna vigintioctomaculata* is distributed in several Asian countries. The larvae and adults often cause substantial economic losses to Solanaceae crops such as potato, tomato, eggplant, and Chinese boxthorn. Even though a chromosome-level genome has been documented, the expression profiles of genes involved in development are not determined. In this study, we constructed embryonic, larval, pupal, and adult transcriptomes, generated a comprehensive RNA-sequencing dataset including ~52 Gb of clean data, and identified 602,773,686 cleaned reads and 33,269 unigenes. A total of 18,192 unigenes were successfully annotated against NCBI nonredundant protein sequences, Swissprot, Eukaryotic Orthologous Groups, Gene Ontology (GO), or Kyoto Encyclopedia of Genes and Genomes (KEGG) databases. There were 3580, 2040, 5160, 2496, 3008, and 3895 differentially expressed genes (DEGs) between adult/egg, egg/larval, larval/pupal, adult/pupal, egg/pupal, and adult/larval samples, respectively. GO and KEGG analyses of the DEGs highlighted several critical pathways associated with specific developing stages. This is the first comprehensive transcriptomic dataset encompassing all developmental stages in *H. vigintioctomaculata*. Our data may facilitate the exploitation of gene targets for pest control and can serve as a valuable gene resource for future molecular investigations.

## 1. Introduction

*Henosepilachna vigintioctomaculata* (Coleoptera: Coccinellidae), commonly known as the 28-spotted larger potato ladybird, is a destructive pest that attacks Solanaceae crops such as potato, tomato, eggplant, and Chinese boxthorn [1]. Both larvae and adults are capable of feeding on mesophyll, leading to significant damage in agricultural production [2]. The beetle is mainly distributed in several Asian countries, such as northern China, India, Japan, and North Korea [3]. Up to now, a chromosome-level genome of *H. vigintioctomaculata* has been assembled. A comparison of positively selected genes to other ladybirds reveals that *H. vigintioctomaculata* possesses different key gene families associated with environmental adaptation [1]. 

*H. vigintioctomaculata* is a holometabolous insect that undergoes four distinct life stages: egg, larva, pupa, and adult. An understanding of the molecular mechanisms that regulate *H. vigintioctomaculata* metamorphosis may facilitate the exploitation of more sustainable and environmentally friendly pest control approaches. Up to now, nevertheless, the expression profiles of involved genes have not been determined. Transcriptional sequencing technology has become a standard tool in biological research for investigating gene expression profiles and biological molecular evolution [4,5,6,7]. For instance, quantitative and qualitative transcriptome analyses can identify critical genes in different developmental stages in Orthoptera *Acheta domesticus* [8], Hemiptera *Aphis aurantii* [9], Coleoptera *Henosepilachna vigintioctopunctata* [10], *Tribolium castaneum* [11], *Cylas formicarius* [12], Diptera *Drosophila melanogaster* [13], *Zeugodacus cucurbitae* [14], *Bactrocera dorsalis* [15], Lepidoptera *Dendrolimus houi* [16], and Hymenoptera *Formica fusca* [17]. In addition, gene expression has been studied by RNA-sequencing (RNA-seq) to identify tissue-specific genes involved in insect reproduction in *Copris tripartitus*, *B. dorsalis* [18,19], *Rhynchophorus ferrugineus* [20], and *Henosepilachna vigintioctopunctata* [21]. Here, Yang et al. emphasized the importance of conducting transcriptomic analyses separately for males and females due to the differences in their reproductive systems, in order to obtain more precise results [20]. Thus, RNA-seq technology allows us to determine critical genes to underlie certain biological functions and identify potential targets for new environmentally friendly insecticides.

In the present paper, RNA-seq was conducted on samples from four stages of *H. vigintioctomaculata* with three biological replicates. We used RNA-seq alignments produced from HISAT2 to perform genome-guided assembly by StringTie v2.1.6 [22,23]. We uncovered several critical pathways associated with specific developing periods. This useful resource will aid us in discovering stage-specific control strategies.

## 2. Results

### 2.1. Data Description and Processing

**Figure 1:**
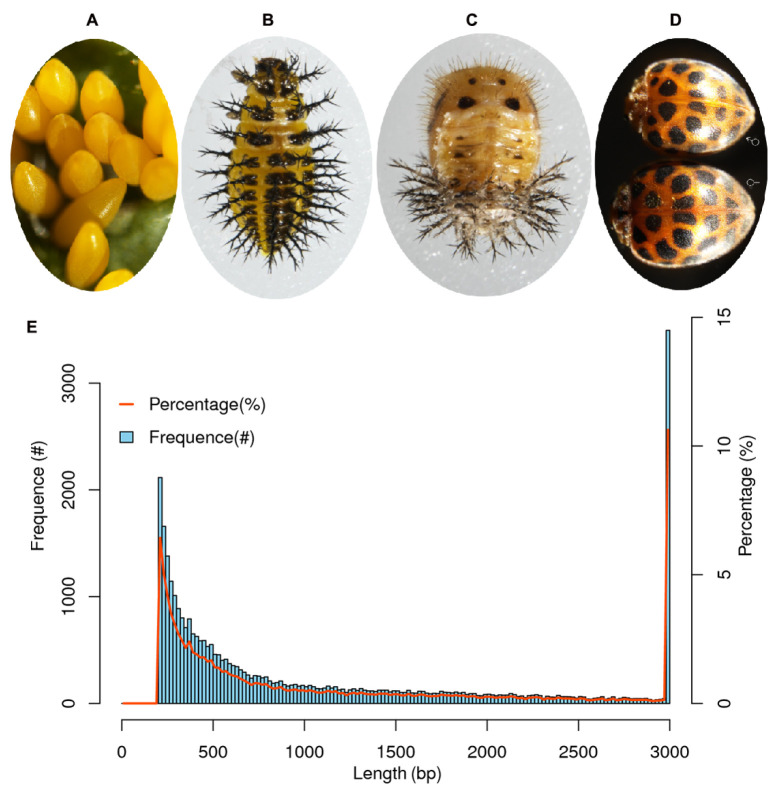
Four developing stages of *Henosepilachna vigintioctomaculata* and length distribution of unigenes. (**A**). egg; (**B**). larva; (**C**). pupa; (**D**). adult; (**E**). the left and right *x*-axes represent the length and percentage of unigenes; the *y*-axis indicates the number of unigenes.

To characterize the expression dynamic of genes throughout development, we conducted RNA-seq experiments using RNA specimens isolated from 12 whole-animal samples representing 4 distinct development stages: egg, larva, pupa, and adult (Figure 1A–D). Illumina Hiseq technology was employed to comprehensively sequence the transcriptomes (Table S1). A total of 611,511,562 reads were obtained, and 602,773,686 clean reads were obtained and used for *de novo* assembly, which accounted for 98.57% of the respective raw reads. The mean Q20 and Q30 were 96.67 and 91.49, respectively. The GC content in all samples ranged from 36.03–39.54%, with an average of 38.24% (Table S1). 

After *de novo* assembly based on the clean reads, the nucleotides of the combined reads for the 12 samples were 42,674,358 bp, and a total of 33,269 unigenes (transcripts) were obtained. The length of the transcripts varied from 201 to 41,072 bp, with an average length of 1255 bp and an N50 length of 2475 bp. A total of 22,290 (67%) and 14,971 (45%) transcripts exceeded 500 and 1000 bp in length (Table S2, Figure 1E).

### 2.2. Function Annotation

**Figure 2:**
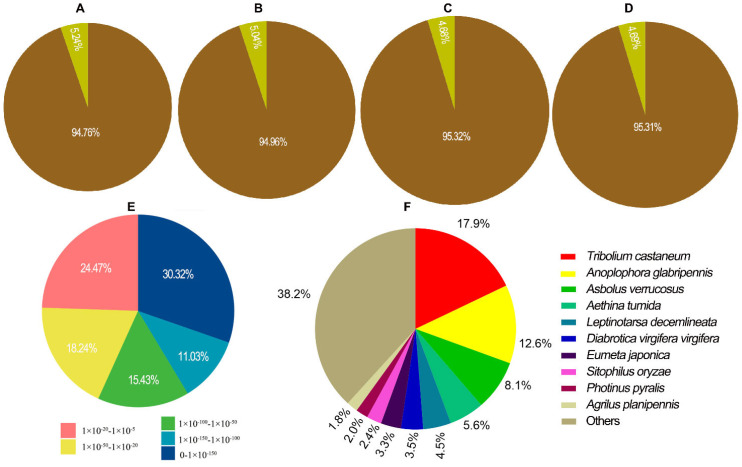
The total mapped rates of the transcriptome reads to the genomic data of *H. vigintioctomaculata* and most closely related insect species. (**A**). egg; (**B**). larva; (**C**). pupa; (**D**). adult; (**E**,**F**). the distribution of E-values and resulting from annotation with the nr database.

We performed alignment analysis based on the reference genomes using the HISAT2 software. Compared with the *H. vigintioctomaculata* genome data, the total mapped rates of the egg, larval, pupal, and adult transcriptome reads reached 94.76%, (Figure 2A), 94.96% (Figure 2B), 95.32% (Figure 2C), and 95.31% (Figure 2D), respectively. 

The *H. vigintioctomaculata* genome data were also compared with those in the NR protein database. Around 56.7% of the transcripts exhibited perfect matches with available sequences (E-value < 1.0 × 10^−50^), while the remaining 43.3% showed matches with an E-value between 0 and 1.0 × 10^−50^ (Figure 2E). Species distribution analysis of the top blast hits in the NR database indicated that 3237 transcripts had the highest homology with *Tribolium castaneum* (17.9%), followed by *Anoplophora glabripennis* (12.6%), *Asbolus verrucosus* (8.1%), *Aethina tumida* (5.6%), and *Leptinotarsa decemlineata* (4.5%) (Figure 2F).

All distinct sequences were annotated against five databases, and a total of 18,192 distinct genes (54.7%) were annotated, which means that a gap in knowledge is still present regarding the genetic information of this species. In total, 18,082 (54.35%), 8507 (25.57%), 8790 (27.42%), 14,724 (44.26%), and 15,453 (46.45%) genes were mapped to NCBI nonredundant protein sequences (Nr), Swissprot, Eukaryotic Orthologous Groups (KOG), Gene Ontology (GO), and Kyoto Encyclopedia of Genes and Genomes (KEGG), respectively (Table S3).

**Figure 3:**
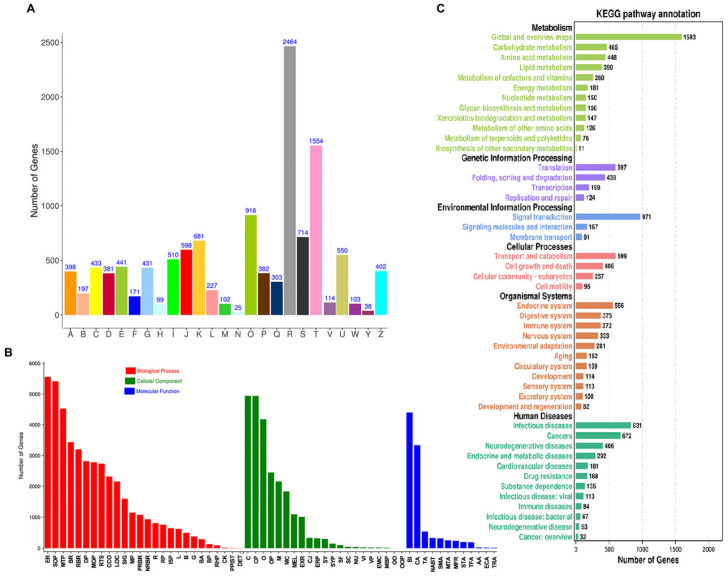
Function annotation of *H. vigintioctomaculata* transcripts using KOG (**A**), GO (**B**), and KEGG (**C**). For COG, the name for each class definition is also provided. A, RNA processing and modification; B, Chromatin structure and dynamics; C, Energy production and conversion; D, Cell cycle control, cell division, chromosome partitioning; E, Amino acid transport and metabolism; F, Nucleotide transport and metabolism; G, Carbohydrate transport and metabolism; H, Coenzyme transport and metabolism; I, Lipid transport and metabolism; J, Translation, ribosomal structure, and biogenesis; K, Transcription; L, Replication, recombination and repair; M, Cell wall/membrane/envelope biogenesis; N, Cell motility; O, Posttranslational modification, protein turnover, chaperones; P, Inorganic ion transport and metabolism; Q, Secondary metabolites biosynthesis, transport and catabolism; R, General function prediction only; S, Function unknown; T, Signal transduction mechanisms; U, Intracellular trafficking, secretion and vesicular transport; V, Defense mechanisms; W, Extracellular structures; Y, Nuclear structure; Z, Cytoskeleton. For GO, AA, antioxidant activity; B, behavior; BA, biological adhesion; BI, binding; BP, biological phases; BR, biological regulation; C, cell; CA, catalytic activity; CCO, cellular component organization or biogenesis; CJ, cell junction; CK, cell killing; CP, cell part; DET; detoxification; DP, developmental process; ECA, electron carrier activity; EMC, extracellular matrix component; ER, extracellular region; ERP, extracellular region part; EXR, extracellular region; G, growth; ISP, immune system process; L, locomotion; LOC, localization; M, membrane; MBP, membrane part; MC, macromolecular complex; MEL, membrane-enclosed lumen; MFR, molecular function regulator; MOP, multicellular organismal process; MP, multi-organism process; MTA, molecular transducer activity; MTP, metabolic process; NABT, nucleic acid binding transcription factor activity; NRBR, negative regulation of biological process; NU, nucleoid; O, organelle; OO, other organism; OOP, other organism part; OP, organelle part; PPIST, presynaptic process involved in synaptic transmission; PRBR, positive regulation of biological process; R, reproduction; RBR, regulation of biological process; RHP, rhythmic process; RP, reproductive process; RTS, response to stimulus; SC, supramolecular complex; SF, supramolecular fiber; SIG, signaling; SMA, structural molecule activity; SOP, single-organism process; STA, signal transducer activity; SY, synapse; SYP, synapse part; TA, transporter activity; TFA, transporter factor activity (protein binding); TRA, transporter regulator activity; VI, virion; VP, virion part.

KOG-based annotation assigned these transcripts to 25 different functional categories (Figure 3A). The largest KOG category was the general function prediction-only category (2464 transcripts). The following abundant groups were signal transduction mechanisms (1554 transcripts), posttranslational modification, protein turnover, and chaperones (916 transcripts), function unknown (714 transcripts), transcription (681 transcripts), and translation, ribosomal structure, and biogenesis (598 transcripts). The three groups involving coenzyme transport and metabolism (99 transcripts), nuclear structure (38 transcripts), and cell motility (25 transcripts) exhibited the smallest KOG classifications (Figure 3A).

The annotated transcripts were classified into three GO categories: biological process (26 GO terms), cellular component (21 GO terms), and molecular function (12 GO terms) (Figure 3B). The top three terms in the biological process were cellular process, single-organism process, and metabolic process, and in the cellular component category, we observed cells, cell parts, and organelles. In the molecular function category, the most enriched terms were binding, catalytic activity, and transporter activity (Figure 3B).

In order to further functional classification and annotation of these transcripts, 14,724 sequences were annotated with the KEGG database. The results showed that the transcripts were distributed among 344 KEGG pathways. Among them, metabolic pathways had the largest number. The top three pathways were global and overview maps (1593), carbohydrate metabolism (465), and amino acid metabolism (448), whereas biosynthesis of other secondary metabolites (11) was the smallest group (Figure 3C).

### 2.3. Differential Expression Genes

**Figure 4:**
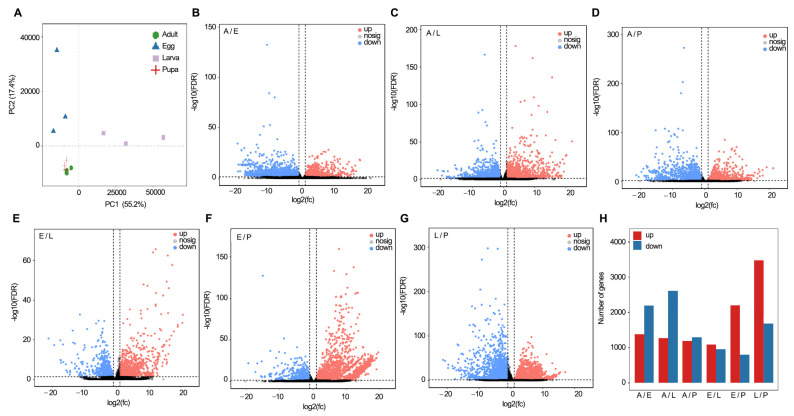
Comparison of transcriptome profiles of all developmental stages in *H. vigintioctomaculata*. (**A**). principal coordinate analysis (PCoA) of samples; (**B**–**G**). volcano plot of gene expression profiles; (**H**). the number of up- and down-regulated genes.

PCoA analysis showed that egg and larval samples were located in the different areas of the graph, whereas the samples of pupal and adult groups partially overlapped (Figure 4A). This implies that the expressed genes in the egg and larval groups are significantly different from the pupal and adult specimens. In contrast, the actively transcribed genes between egg/larva and pupa/adult show higher similarity (Figure 4A).

The differential expression genes (DEGs) (fold change ≥ 2.0 and *p* < 0.01) were compared (Figure 4B–G). Consistent with the result shown in Figure 4A, DEGs concerning adult/egg, egg/pupal, larval/pupal, and adult/larval samples were 3580 (1381↑/2199↓), 3008 (2208↑/800↓), 5160 (3477↑/1683↓), and 3895 (1276↑/2619↓) respectively, whereas DEGs concerning egg/larval and adult/pupal specimens were 2040 (1088↑/952↓) and 2496 (1195↑/1301↓), respectively (Figure 4B, Table S4). Both PcoA analysis and DEG data are in accordance with a common notion that the transcriptomes in the juvenile stages (embryo, larva) are dramatically different from those in the adultoid (pupa) and adult periods (Figure 4H).

### 2.4. Adult versus Egg

**Figure 5:**
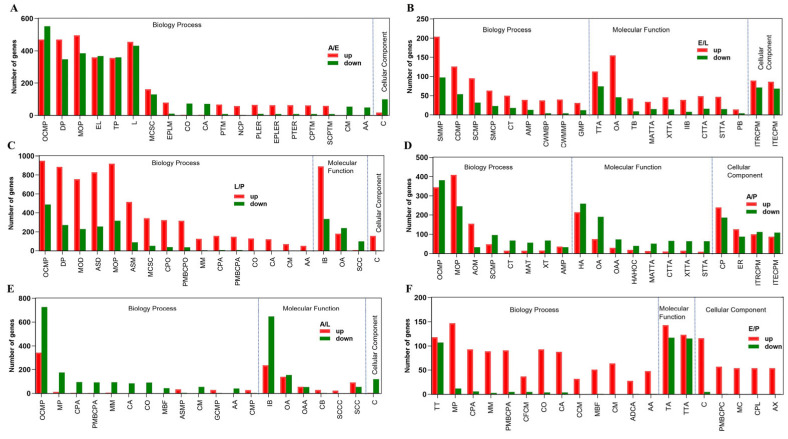
The representative GO terms enriched by differently expressed genes during different stages. (**A**). adult versus egg (A/E); (**B**). adult versus larva (A/L); (**C**). adult versus pupa; (**D**). egg versus larva (E/L); (**E**). egg versus pupa (E/P); (**F**). larva versus pupa (L/P). AA, axoneme assembly; ADCA, axonemal dynein complex assembly; AMP, aminoglycan metabolic process; AOM, animal organ morphogenesis; ASD, anatomical structure development; ASMP, amino sugar metabolic process; AX, axoneme; C, cilium; CA, cilium assembly; CB, chitin binding; CCM, cilium-dependent cell motility; CDMP, carbohydrate derivative metabolic process; CFCM, cilium or flagellum-dependent cell motility; CM, cilium movement; CMP, chitin metabolic process; CO, cilium organization; CP, cell periphery; CPL, ciliary plasm; CPO, cell projection organization; CPA, cell projection assembly; CPO, cell projection organization; CPTM, cotranslational protein targeting to membrane; CT, carbohydrate transport; CTTA, carbohydrate transmembrane transporter activity; CWMBP, cell wall macromolecule biosynthetic process; CWMMP, cell wall macromolecule metabolic process; EL, establishment of localization; EPLER, establishment of protein localization to endoplasmic reticulum; EPLM, establishment of protein localization to membrane; GCMP, glucosamine-containing compound metabolic process; GMP, glycosaminoglycan metabolic process; HA, hydrolase activity; HAHOC, hydrolase activity, hydrolyzing O-glycosyl compounds; IB, ion binding; IIB, iron ion binding; ITECPM, integral component of plasma membrane; L, localization; ITRCPM, intrinsic component of plasma membrane; MAT, monocarboxylic acid transport; MATTA, monocarboxylic acid transmembrane transporter activity; MCSC, movement of cell or subcellular component; MBF, microtubule bundle formation; MC, motile cilium; MM, microtubule-based movement; MOD, multicellular organism development; MP, microtubule-based process; NCP, nuclear-transcribed mRNA catabolic process, nonsense-mediated decay; OA, oxidoreductase activity; OAA, oxidoreductase activity, acting on CH-OH group of donors; OCMP, organonitrogen compound metabolic process; PB, pigment binding; PLER, protein localization to endoplasmic reticulum; PMBCPA, plasma membrane bounded cell projection assembly; PMBCPC, plasma membrane bounded cell projection cytoplasm; PMBCPO, plasma membrane bounded cell projection organization; PTER, protein targeting to ER; PTM, protein targeting to membrane; SCC, structural constituent of cuticle; SCCC, structural constituent of chitin-based cuticle; SCMP, sulfur compound metabolic process; SCPTM, SRP-dependent cotranslational protein targeting to membrane; SMCP, small molecule catabolic process; SMMP, small molecule metabolic process; STTA, sugar transmembrane transporter activity; TB, tetrapyrrole binding; TP, transport; TT, transmembrane transport; TTA, transmembrane transporter activity; XT, xenobiotic transport; XTTA, xenobiotic transmembrane transporter activity. Other abbreviations are given in Figure 3.

GO enrichment of DEGs for the adult/egg group displayed that 19 out of the top 20 enriched belong to the biological process category. The top five are organo-nitrogen compound metabolic process, developmental process, multicellular organismal process, establishment of localization, and transport (Figure 5A).

**Figure 6:**
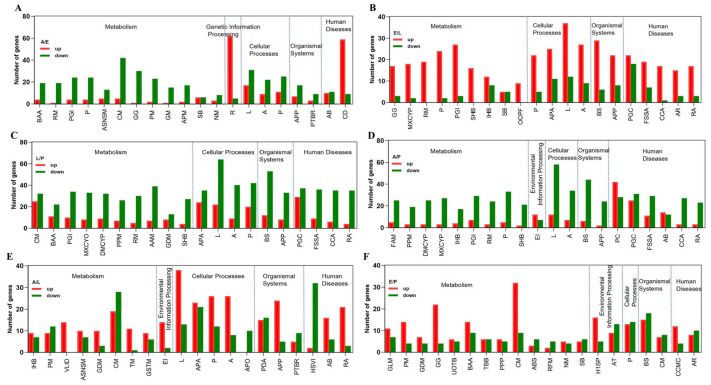
The representative KEGG pathway enriched by differently expressed genes during different stages. (**A**). adult versus egg (A/E); (**B**). adult versus larva (A/L); (**C**). adult versus pupa; (**D**). egg versus larva (E/L); (**E**). egg versus pupa (E/P); (**F**). larva versus pupa (L/P). BAA, Biosynthesis of amino acids; RM, Retinol metabolism; PGI, Pentose and glucuronate interconversions; P, Phagosome; ASNSM, Amino sugar and nucleotide sugar metabolism; CM, Carbon metabolism; GG, Glycolysis/Gluconeogenesis; PM, Pyruvate metabolism; OCPF, One carbon pool by folate; GM, Galactose metabolism; APM, Arginine and proline metabolism; SB, Steroid biosynthesis; UOTB, Ubiquinone and other terpenoid-quinone biosynthesis; NM, Nitrogen metabolism; MXCYP, Metabolism of xenobiotics by cytochrome P450; IHB, Insect hormone biosynthesis; DMCYP, Drug metabolism—cytochrome P450; GDM, Glyoxylate and dicarboxylate metabolism; VLID, Valine, leucine and isoleucine degradation; TM, Tryptophan metabolism; GSTM, Glycine, serine and threonine metabolism; FAM, Fatty acid metabolism; PPM, Porphyrin metabolism; SHB, Steroid hormone biosynthesis; GLM, Glycerolipid metabolism; TBB, Terpenoid backbone biosynthesis; PPP, Pentose phosphate pathway; ABS, Arginine biosynthesis; RFM, Riboflavin metabolism; AAM, Ascorbate and aldarate metabolism; R, Ribosome; EI, ECM-receptor interaction; L, Lysosome; APA, Autophagy—animal; APO, Autophagy—other; A, Apoptosis; P, Phagosome; H1SP, HIF-1 signaling pathway; AT, ABC transporters; APP, Antigen processing and presentation; PTBR, Proximal tubule bicarbonate reclamation; PDA, Protein digestion and absorption; BS, Bile secretion; CM, Cholesterol metabolism; AB, Amoebiasis; CD, Coronavirus disease—COVID-19; HSVI, Herpes simplex virus 1 infection; RA, Rheumatoid arthritis; PS, Pathways in cancer; CCMC, Central carbon metabolism in cancer; PGC, Proteoglycans in cancer; FSSA, Fluid shear stress and atherosclerosis; CCA, Chemical carcinogenesis—DNA adducts; AR, Antifolate resistance.

KEGG enrichment of DEGs for the adult/egg group exhibited that 12 among the top 20 enriched are classified into the metabolism category. Pathways related to amino acid biosynthesis, retinol, pyruvate and carbon metabolism, pentose and glucuronate interconversions, glycolysis/gluconeogenesis, and phagosome were active. Active ribosome formation belongs to the genetic information processing category (Figure 6A). In the starch and sucrose metabolism pathway, genes coding for glucosidase, phosphoglucomutase, glucose-1-phosphate uridyltransferase, glycogen synthase, starch-branching enzyme, glycoside hydrolase, trehalase, and glucosidase were higher in developing eggs. In glycolysis/gluconeogenesis, genes encoding 20 enzymes, such as α-D-glucose-1,6-bisphosphatase, hexokinase, glucose-6-phosphate isomerase, phosphofructokinase, fructose-1,6-biphosphate aldolase, glyceraldehyde 3-phosphate dehydrogenase, phosphoglycerate mutase, glycolytic enolase, pyruvate kinase, and pyruvate dehydrogenase, were higher in eggs than those in adults. Genes in developing eggs coding for phosphoenolpyruvate carboxykinase, the pyruvate dehydrogenase complex, cis-aconitase, oxalosuccinate decarboxylase, 2-oxoglutarate dehydrogenase, and malate dehydrogenase in the citrate cycle were more abundant. Similarly, genes encoding NADH: ubiquinone reductase and the ubiquinol-cytochrome c reductase iron-sulfur subunit in oxidative phosphorylation were actively expressed in eggs. The resulting substrates, NADH and FADH2 from glycolysis and the citrate cycle, are used to generate ATP through electron transport and oxidative phosphorylation in the developing embryos (Table S4).

Phosphofructokinase, gluconolactone, glucose-6-phosphate isomerase, fructose-bisphosphate aldolase, transketolase, and phosphopentomutase genes in the pentose phosphate pathway, uridine-5′-diphos (UDP)-glucuronosyltransferases, UDP-phoglucose pyrophosphorylase, and xylulose kinase genes in pentose and glucuronate interconversions were higher in developing eggs. The resultant pentoses were further transferred to D-ribose and 2-deoxy ribose, two substrates for RNA and DNA biosynthesis. Moreover, acetyl-CoA carboxylase, trans-2-enoyl-CoA reductase, and α-D-glucuronidase genes involved in fatty acid biosynthesis and elongation and Δ24-sterol reductase and sterol O-acyltransferase genes associated with steroid biosynthesis were actively transcribed in developing eggs. Long fatty acids and steroids are the main components of the cellular membrane. Furthermore, dozens of amino acid metabolism-related genes, such as monoamine oxidase, nitric oxide synthase, glutamate dehydrogenase, adenylosuccinate lyase, L-asparaginase, D-aspartate oxidase, 4-aminobutyrate aminotransferase, glutamate synthase, phosphoserine transaminase, D-serine ammonia-lyase, serine hydroxymethyltransferase, threonine ammonia-lyase, phosphoserine aminotransferase, cysteine dioxygenase, lactate dehydrogenase, malate dehydrogenase, and S-adenosylhomocysteine hydrolase, were higher in developing eggs (Table S4).

### 2.5. Egg versus Larvae

GO analysis of DEGs from the egg versus larva group showed that out of the top 20 enriched GO terms, 9, 9, and 2 were in the biological process, molecular function, and cellular component categories (Figure 5B). Within the biological process, the small-molecule metabolic process, the carbohydrate derivative metabolic process, the small-molecule catabolic process, carbohydrate transport, and the aminoglycan metabolic process ranked as the top five terms. Among the molecular functions, transmembrane transporter activity, oxidoreductase activity, tetrapyrrole binding, monocarboxylic acid transmembrane transporter activity, and xenobiotic transmembrane transporter activity were the top five terms.

Out of the top 20 enriched from KEGG analysis of egg/larva DEGs, 9 of them belong to the metabolism category (Figure 6B). The nine KEGG pathways were glycolysis/gluconeogenesis, metabolism of xenobiotics by cytochrome P450, retinol metabolism, phagosome, pentose and glucuronate interconversions, steroid hormone biosynthesis, insect hormone biosynthesis, steroid biosynthesis, and one carbon pool by folate. In the developing eggs, the expression levels of methylenetetrahydrofolate dehydrogenase, formyltetrahydrofolate dehydrogenase, formate-tetrahydrofolate ligase, methenyltetrahydrofolate cyclohydrolase, nicotinamide N-methyltransferase, guanidinoacetate N-methyltransferase, and homocysteine S-methyltransferase in one carbon pool by folate, and of alkaline phosphatase, dihydrofolate reductase, aldose reductase, and phenylalanine hydroxylase in folate biosynthesis were richer (Table S4).

As a defoliating pest, *H. vigintioctomaculata* larvae have to degrade chlorophyll from the host plant. As a result, tetrapyrrole binding and porphyrin metabolism were active (Figure 6B). The related genes coding for HemA-HemH, HemJ, HemL, HemN, HemY, CysG, CbiA-CbiH, CbiJ-CbiL, CbiP, CbiT, CbiX, CobA-CobD, CobF-CobL, CobM, CobO, CobQ, CobS, CobU, CobV, CobNST, BtuR, BzaAB, and BzaF were abundantly expressed in the larvae. Moreover, several cyp genes involved in the degradation of xenobiotics were abundantly expressed. Similarly, the expression of amylase, digestive cysteine proteinase, serine protease, peptidase, and lipase was enhanced for the digestion of starch, protein, and triglyceride, while the transcription of genes encoding the SR-B1 protein and PepT for the transmembrane of fatty acids and dipeptide was increased. As a result, glycerophospholipid biosynthesis was active in the larva. Genes encoding glyceraldehyde-3-phosphate dehydrogenase, lysophosphatidyl acyltransferase, platelet-activating factor acetylhydrolase, and lysophospholipase were abundantly transcribed (Table S4). 

Finally, juvenile hormone (JH) and 20-hydroxyecdysone (20E) metabolism pathways were active in both developing egg and larval specimens (Figure 6B). For JH metabolism, the biosynthesis genes encoding farnesol phosphatase, farnesol dehydrogenase, farnesal dehydrogenase, epoxidase, and JH acid O-methyltransferase and the degradation genes coding for JH esterase and JH epoxide hydrolase were abundantly transcribed. For 20E synthesis, genes encoding CYP307A1, CYP315A1, and CYP314A1 were actively expressed (Table S4).

### 2.6. Larva versus Pupa

GO enrichment of larva/pupa DEGs showed that 16, 3, and 1 out of the top 20 enriched GO terms were categorized into the biological process, molecular function, and cellular component classes, respectively (Figure 5C). In the 16 biological process terms, the organonitrogen compound metabolic process, the developmental process, multicellular organism development, anatomical structure development, the multicellular organismal process, anatomical structure morphogenesis, movement of the cell or subcellular component, cell projection organization, and plasma membrane-bounded cell projection organization were the top 10. The three molecular function terms were ion binding, oxidoreductase activity, and structural constituent of the cuticle.

KEGG analysis of larva/pupa DEGs illustrated that 10, 4, 2, and 4 out of the top 20 enriched were sub-classified into the metabolism, cellular process, organismal system, and human diseases categories (Figure 6C). Among the biological processes were cilium movement, biosynthesis of amino acids, pentose and glucuronate interconversions, metabolism of xenobiotics by cytochrome P450, drug metabolism (cytochrome P450), porphyrin metabolism, retinol metabolism, ascorbate and aldarate metabolism, glyoxylate and dicarboxylate metabolism, and steroid hormone biosynthesis.

Similar to developing eggs, active catabolism of carbohydrates in pupae occurs through glycolysis/gluconeogenesis and the citrate cycle. The resulting substrates, NADH and FADH2, are used to generate ATP through electron transport and oxidative phosphorylation. Similarly, the active pentose phosphate pathway and pentose and glucuronate interconversions caused several pentoses, which were further transferred to D-ribose and 2-deoxy ribose for the biosynthesis of RNA and DNA (Table S4). 

Compared to pupa, chlorophyll degradation (porphyrin metabolism)-related genes were up-regulated in *H. vigintioctomaculata* larvae (Figure 6C). Several *cyp* genes involved in the degradation of xenobiotics were abundantly expressed in the larvae. Similarly, the expression of *maltase*, *serine protease*, *carboxypeptidase B*, and *lipase* was enhanced for the digestion of starch, protein, and triglyceride, while the transcription of genes encoding the ABCG8 protein and PepT for the transmembrane of fatty acids and dipeptide was increased (Table S4).

### 2.7. Adult versus Pupa

GO enrichment of adult/pupa DEGs exhibited that 8, 8, and 4 out of the top 20 GO terms were sub-divided into the biological process, molecular function, and cellular component categories, respectively (Figure 5D). The eight molecular function terms were hydrolase activity, oxidoreductase activity, oxidoreductase activity (acting on CH-OH group of donors), hydrolase activity (hydrolyzing O-glycosyl compounds), monocarboxylic acid transmembrane transporter activity, carbohydrate transmembrane transporter activity, xenobiotic transmembrane transporter activity, and sugar transmembrane transporter activity.

The KEGG enrichment pathway from adult/pupa DEGs displayed that 9, 1, 2, 2, and 6 out of the top 20 enriched were divided into the metabolism, environmental information processing, cellular process, organismal system, and human diseases groups, respectively (Figure 6D). Among the nine metabolism terms, there were fatty acid metabolism, porphyrin metabolism, drug metabolism (cytochrome P450), metabolism of xenobiotics by cytochrome P450, insect hormone biosynthesis, pentose and glucuronate interconversions, retinol metabolism, phagosome, and steroid hormone biosynthesis.

A high pulse of 20E triggers pupa–adult ecdysis. The biosynthesis of the steroid hormone was an important biochemical pathway in the pupal stage (Figure 6D). Consistently, the genes encoding steroid hormone generation enzymes such as CYP (EC 1.14.14.1), estradiol 17β-dehydrogenase, and UDP-glucuronosyltransferase were higher in the pupae (Table S4).

Compared with pupae, adults actively ingest plant foliage. Therefore, chlorophyll degradation (porphyrin metabolism)-related genes were up-regulated (Figure 6D). Several *cyp* and *myrosinase* genes involved in the degradation of xenobiotics were abundantly expressed. Similarly, the expression of maltase, trypsin-1, carboxypeptidase B, and lipase was enhanced for the digestion of carbohydrates, protein, and triglyceride (Table S4).

### 2.8. Adult versus Larva

In the GO enrichment terms from adult/larva DEGs, 13, 6, and 1 out of the top 20 enriched were categorized as the biological process, molecular function, and cellular component sets, respectively (Figure 5E). Among the six molecular function terms were ion binding, oxidoreductase activity, oxidoreductase activity (acting on the CH-OH group of donors), chitin binding, the structural constituent of the chitin-based cuticle, and the structural constituent of the cuticle.

KEGG enrichment pathways from adult/larva DEGs demonstrated that 8, 1, 5, 3, and 3 out of the top 20 enriched belonged to the metabolism, environmental information processing, cellular process, organismal system, and human diseases categories (Figure 6E). In the metabolism category, we observed insect hormone biosynthesis, pyruvate metabolism, valine, leucine and isoleucine degradation, amino sugar and nucleotide sugar metabolism, glyoxylate and dicarboxylate metabolism, carbon metabolism, tryptophan metabolism, and glycine, serine, and threonine metabolism.

Larvae need JH and 20E for growth and metamorphosis, while adults need the two hormones to regulate oogenesis. Therefore, the insect hormone biosynthesis KEGG pathway ranked first within the metabolism category (Figure 6E). In the larvae, JH synthesis was more active whereas degradation was less active. In contrast, adults biosynthesized more 20E and degraded less (Table S4).

Compared with larvae, adults have thicker cuticles and need more chitin (Figure 6E). Consistently, the expression of *chitin synthase* and *chitinase* was more active in adults. Moreover, the mRNA levels of *dopa decarboxylase* and *3*,*4-dihydroxyphenylacetaldehyde synthase* were higher in adults than those in the larvae. It appears more melanins and sclerotins are formed for the pigmentation and sclerotization of the adult cuticle (Table S4).

### 2.9. Egg versus Pupa

GO enrichment of egg/pupa DEGs showed that 13, 2, and 5 out of the top 20 enriched were sub-classified into the biological process, molecular function, and cellular component categories, respectively (Figure 5F). Under the biological process ontology, transmembrane transport, microtubule-based process, cell projection assembly, microtubule-based movement, plasma membrane-bounded cell projection assembly, cilium or flagellum-dependent cell motility, cilium organization, cilium assembly, cilium-dependent cell motility, and microtubule bundle formation ranked as the top 10. The two molecular function terms were transporter activity and transmembrane transporter activity.

The KEGG enrichment pathway from egg/pupa DEGs revealed that the top 20 enriched were divided into the metabolism, environmental information processing, cellular process, organismal system, and human disease classifications, containing 13, 2, 1, 2, and 2 issues, respectively (Figure 6F). The 13 metabolism terms were glycerolipid metabolism, pyruvate metabolism, glyoxylate and dicarboxylate metabolism, glycolysis/gluconeogenesis, ubiquinone and other terpenoid-quinone biosyntheses, biosynthesis of amino acids, terpenoid backbone biosynthesis, pentose phosphate pathway, carbon metabolism, arginine biosynthesis, riboflavin metabolism, nitrogen metabolism, and steroid biosynthesis.

The comparison of egg and pupal samples for KEGG metabolism terms revealed that eggs mainly depended on the catabolism of carbohydrates to generate ATP, whereas pupae mainly applied fatty acids as the energy source (Table S4).

## 3. Discussion

Our goal in the current paper was to obtain life-stage-specific expression patterns and annotate key genes that could be vital for metamorphosis. Compared with the genomic data [1], the total mapped rates in egg, larval, pupal, and adult transcriptome reads reached 94.76%, 94.96%, 95.32%, and 95.31%, respectively. Therefore, our transcriptome data are reliable. Our results highlighted several critical pathways associated with specific developing stages. Accordingly, we tried to search for potential genes for developing stage-specific control strategies in *H. vigintioctomaculata*.

### 3.1. Potential Targets for Control throughout the Whole Lifespan

Typical holometabolous insects undergo hatching, molting, pupation, and emergence during their life cycles. Each of these developmental processes is tightly regulated by two hormones, 20E and JH [24,25,26]. Disruption of either JH [27,28] or 20E signaling [29,30,31,32,33] causes lethality, impairs growth, or inhibits reproduction. In *Tribolium castaneum*, following hatching, the expression of the juvenile hormone (JH) and ecdysteroid biosynthesis genes decreased, while the expression of JH degradation genes increased after pupation and eclosion. Additionally, the ecdysteroid degradation gene CYP18A1 exhibited a decrease in expression following pupation [11]. In the present paper, we found that insect hormone biosynthesis or steroid hormone biosynthesis KEGG terms were among the most preferential words in all developing stages. Therefore, the JH and 20E signaling genes are the potential targets for controlling *H. vigintioctomaculata* throughout the whole lifespan.

During molting, pupation, and emergence, insects have to replace their old integument with a newly formed cuticle. Thus, genes in chitin metabolism pathways are expressed dynamically to keep pace with insect growth and metamorphosis [34]. Therefore, chitin synthases and chitinases are vital for metamorphosis. Knockdown of chitin biosynthesis genes impairs ecdysis in beetles [35,36,37,38]. Similarly, RNA interference (RNAi) for chitinase genes causes severe defects in developing insects in *Tribolium castaneum* [39], *Plutella xylostella* [40], *Chilo suppressalis* [41], and *Nilaparvata lugens* [42]. In the present study, KEGG enrichment of DEGs revealed different expression patterns of chitin synthase and chitinase genes among development, suggesting critical roles of chitin metabolism in different developing stages in *H. vigintioctomaculata*.

After ecdysis, the new cuticle must be tanned to provide protection against physical injury and water loss, rigidness for muscle attachment and mechanical support, and flexibility in intersegmental and joint areas for mobility [43]. From a biochemical perspective, the tanning of insect cuticles results from a combination of melanins and sclerotins [44]. Thus, RNAi of the pigment formation pathway genes inhibits cuticle hardening and results in beetle lethality [43,44,45,46]. In this study, our data revealed that several pigment synthesis genes, such as dopa decarboxylase and 3,4-dihydroxyphenylacetaldehyde synthase, were actively expressed in specific developing stages. All these results suggest potential targets for controlling *H. vigintioctomaculata*. It is worth noting that, in this study, mixed samples of larvae from different instar stages were used. Due to the physiological and biochemical differences that exist between larvae at different instar stages, the use of mixed samples may have certain limitations, and further investigation into the differences between larvae at different instar stages may be necessary in future studies.

### 3.2. Targeting Energy Generation Genes in Embryonic and Pupal Stages

Given that eggs initiate embryogenesis until hatching after deposition by female adults [47], we collected 3-day-old eggs that were undergoing embryogenesis. We discovered that the processes in embryos and pupae were mostly associated with energy production and biosynthesis for proteins, RNA, and DNA. In accordance with our data, a D. melanogaster embryo develops into a larva with approximately 10^5^ cells in 24 h at room temperature, utilizing 10 mJ of energy generated by the oxidation of the maternal glycogen and triacylglycerol stores [47]. 

Any strategies that can inhibit the obtainment of ATP may kill developing embryos or pupae. Dysfunction of the respiratory chain can hinder ATP production and elevate ROS generation and consequently leads to a deficiency in respiratory chain complex proteins [48]. A series of respiratory agents, such as rotenone, ptericidin, Amytal, and other barbiturates, mercurial agents, Demerol, 2-ternoyltrifluoroacetone, carboxin, Antimycin, cyanide, azide, carbon monoxide, oligomycin, and organic arsenical chemicals, can exert inhibitory effects on the respiratory chain [48,49,50]. 

The inhibition of the entry of oxygen into eggs and pupae is another pathway. For instance, Drosophila embryogenesis arrests in response to hypoxia [51]. In this context, mineral oil acting to block the entry of oxygen can be used as an insecticide or synergist against agricultural pests [52,53] or against insect vectors to protect plants from virus infections [54]. Discovering effective insecticides that can completely inhibit the entry of oxygen into eggs and pupae but are safe for the host plant is a challenge and deserves further research. Moreover, RNAi-aided knockdown of genes involved in ATP generation is a potential control strategy in *H. vigintioctomaculata*.

### 3.3. Antifeedants for Larvae and Adults

To date, the two environmentally friendly control methods for beetles are (1) the conservation and augmentation of indigenous natural enemies and (2) the use of double-stranded RNA (dsRNA) for RNAi [45]. In order to improve the compatibility of these two promising approaches, a dsRNA-based pesticide should act as an antifeedant to reduce leaf damage and as a slower-acting insecticide that can keep pests alive for a substantial time period to attract and arrest predators and parasitoids [45]. Our recent endeavor identifies a p-diphenol:dioxygen oxidoreductase laccase 2 gene Hvlac2 as a suitable dsRNA target gene to improve the compatibility of the two environmentally friendly control approaches in *H. vigintioctopunctata* [45]. Yang et al. emphasized the importance of conducting separate transcriptomic analyses for male and female adults due to the differences in their reproductive systems, in order to obtain more accurate results. However, this study analyzed male and female adults collectively, which may introduce certain limitations when investigating reproductive-related pathways. Further refinement and categorization will be necessary in future studies [20].

In the current paper, our data showed that a subset of genes related to food digestion and nutrient absorption were actively expressed in larvae and adults. Our findings provided potential targets to develop RNAi-based antifeedants for compatible control strategies to *H. vigintioctomaculata*.

## 4. Materials and Methods

### 4.1. Insect Rearing

*H. vigintioctomaculata* used for this study was kindly provided by Dr. Guo Wen-Chao in Xinjiang Academy of Agricultural Sciences, which was collected from *Solanum melongena* L. in Urumqi, Xinjiang, China in 2021. The larvae were routinely maintained in an insectary at 25 ± 1 °C under a 16 h: 8 h light–dark photoperiod and 50–60% relative humidity on potato foliage at vegetative growth or tuber initiation stages.

### 4.2. Sample Collection

The 3-day-old eggs, larvae (including first-, second-, third-, and fourth-instar larvae), 2-day-old pupae, and 5-day-old adults of *H. vigintioctomaculata* were sampled. The number of individuals for each replicate across the different developmental stages was as follows: 200 eggs, 20 first- and second-instar larvae, 10 third- and fourth-instar larvae, 5 pupae, and 2 adults (1 male and 1 female). The collection was repeated three times.

### 4.3. Total RNA Isolation

Total RNA was extracted from the *H. vigintioctomaculata* using TRIzol reagent (Invitrogen, New York, NY, USA) following the manufacturer’s protocol. The RNA concentration was examined using a NanoDrop 2000 spectrophotometer (Thermo Fisher Scientific, New York, NY, USA). The purity of RNA was assessed by measuring the absorbance ratios at OD260/280 and OD260/230. The integrity of RNA was evaluated by 1% agarose gel electrophoresis with ethidium bromide staining.

### 4.4. Library Construction and Transcriptomic Sequencing

The transcriptome libraries were constructed using the NEBNext UltraTM RNA Library Prep Kit for Illumina (NEB, San Diego, CA, USA) following the standard manufacturer’s instructions. Briefly, 1.5 µg total RNA in each sample was used for mRNA enrichment using poly-T oligo-attached magnetic beads. Fragmentation into 300~350 bp sequences was conducted using divalent cations under elevated temperatures in NEBNext First Strand Synthesis Reaction Buffer. The first-strand cDNA was reverse-transcribed using fragmented RNA and dNTPs (dATP, dTTP, dCTP, and dGTP) and second-strand cDNA synthesis was subsequently performed using the NEBNext FirstStrand Synthesis kit. The remaining overhangs of double-strand cDNA were converted into blunt ends via exonuclease/polymerase activity. After the adenylation of the 3′ ends of DNA fragments, sequencing adaptors were ligated to the cDNA and the library fragments were purified. The template was enriched using Phusion High-Fidelity DNA polymerase (Thermo Scientific, MA, USA), Universal PCR primers, and the Index (X) Primer. The PCR product was purified (AMPure XP system, CA, USA) and the quality was re-assessed on the Agilent Bioanalyzer 2100 system.

### 4.5. Sequencing Processing and Assembly

After library preparation and the pooling of different samples, the specimens were subjected to Illumina sequencing using lncRNA-seq PE150 (paired-end 150 nt) sequencing. Raw data (raw reads) of the FASTQ format were first processed using in-house perl scripts. In this step, clean data (clean reads) were obtained by removing the following reads: (1) reads with an adapter; (2) reads with more than 3 N; (3) and reads with more than 20% nucleotides with a Qphred value of less than 5. At the same time, the Q20, Q30, and GC content of the clean data was calculated. Then, the rRNA was removed by mapping the clean reads to the Silva database. RNA-seq raw data were deposited into the Genebank database with the accession number PRJNA789715.

All the downstream analyses were based on clean data without rRNA. The clean reads were aligned to the reference genome of *H. vigintioctomaculata* obtained from the CNCB database using Hisat2 with default parameters [1,22].

### 4.6. Identification of Differentially Expressed Genes

EdgeR was used for differential expression analysis [55]. The resulting *p*-values were adjusted using the Benjamini and Hochberg approach to controlling the false discovery rate. Genes with |log2 (Fold Change)| > 1 and q value < 0.05 were assigned as differentially expressed.

### 4.7. Transcript Data Annotation and Gene Ontology

The topG http://www.bioconductor.org/packages/release/bioc/html/topGO.html (accessed on 20 January 2024) was used to obtain GO (Gene Ontology) terms with an E-value < 10^−5^. KEGG (Kyoto Encyclopedia of Genes and Genomes) enrichment analysis of differentially expressed gene sets was implemented using the KOBAS package [56]. The Unigene function was also annotated based on Nr (NCBI nonredundant protein sequences), Swiss-Prot (A manually annotated and reviewed protein sequence database), and KOG/COG (Clusters of Orthologous Groups of proteins).

## 5. Conclusions

This study employs high-throughput sequencing to analyze the transcriptomes at various developmental stages (egg, larva, pupa, and adult) of *H. vigintioctomaculata*. These findings may facilitate the exploitation of gene targets for pest control. Moreover, our data significantly enhanced the understanding of the genomic resources and can serve as a foundation for investigating the developmental and reproductive variations in *H. vigintioctomaculata*, as well as other beetles.

## Data Availability

Data generated in association with this study are available in the Supplementary Materials published online with this article.

## Supplementary Materials

The following supporting information can be downloaded at: https://www.mdpi.com/article/10.3390/ijms25147505/s1.
