# Supplementary material for: Comparative Transcriptome Analysis of Henosepilachna vigintioctomaculata Reveals Critical Pathways during Development
Source: Int J Mol Sci. 2024 Jul 9;25(14):7505. doi: 10.3390/ijms25147505 (PMC11276636; doi:10.3390/ijms25147505)
Supplement: Supplementary file 1 [file ijms-25-07505-s001.zip › Table S1.pdf]

Table S1 Summary of data before and after raw reads filtering

Sample	Raw reads	Cleaned reads	Cleaned reads%	RawData(bp)	Q20(%)	Q30(%)	GC(%)
Egg-1	55253164	54307662	98.29	8287974600	96.11	90.11	37.60
Egg-2	63420902	62529934	98.60	9513135300	96.99	92.21	36.03
Egg-3	47101640	46237844	98.17	7065246000	96.13	90.40	37.87
Larva-1	45986362	45384900	98.69	6897954300	97.16	92.62	39.37
Larva-2	48265666	47659462	98.74	7239849900	97.09	92.45	39.14
Larva-3	54341050	53636000	98.70	8151157500	96.95	92.08	39.54
Pupa-1	45089486	44430970	98.54	6763422900	96.49	91.10	38.30
Pupa-2	54899114	54174762	98.68	8234867100	96.95	92.08	37.69
Pupa-3	60360666	59501096	98.58	9054099900	96.75	91.96	38.36
Adult-1	45942980	45307734	98.62	6891447000	96.30	90.39	38.82
Adult-2	47405884	47005506	99.16	7110882600	97.15	92.31	38.55
Adult-3	43444648	42597816	98.05	6516697200	96.00	90.14	37.63
All	611511562	602773686	98.57	91726734300	96.67	91.49	38.24
