# Supplementary material for: Comparative Transcriptome Analysis of Henosepilachna vigintioctomaculata Reveals Critical Pathways during Development
Source: Int J Mol Sci. 2024 Jul 9;25(14):7505. doi: 10.3390/ijms25147505 (PMC11276636; doi:10.3390/ijms25147505)
Supplement: Supplementary file 1 [file ijms-25-07505-s001.zip › Table S2.pdf]

Table S2 Assembly information of the *H. vigintioctomaculata* transcriptome dataset

Type	Resoure
**Genes Num**	33269
**N50 number**	4825
**N50 length**	2475
**Max length**	41072
**Min length**	201
**Average length**	1255
**Total assembled bases**	42674358
