# Supplementary material for: Comparative Transcriptome Analysis of Henosepilachna vigintioctomaculata Reveals Critical Pathways during Development
Source: Int J Mol Sci. 2024 Jul 9;25(14):7505. doi: 10.3390/ijms25147505 (PMC11276636; doi:10.3390/ijms25147505)
Supplement: Supplementary file 1 [file ijms-25-07505-s001.zip › Table S3.pdf]

Table 3 Annotation information of the *H. vigintioctomaculata* transcriptome dataset

	**Unigene number (%)**
**NR**	18082 (54.35)
**Swiss-Prot**	8507 (25.57)
**KOG**	8790 (27.42)
**KEGG**	14724(44.26)
**GO**	15453(46.45)
**Without annotation gene**	15077 (45.32)
**Total Unigenes**	33269(100)
